# Revealing the Mechanism of Esculin in Treating Renal Cell Carcinoma Based on Network Pharmacology and Experimental Validation

**DOI:** 10.3390/biom14081043

**Published:** 2024-08-22

**Authors:** Zixuan Chen, Cunzhou Wang, Yuesong Cai, An Xu, Chengtao Han, Yanjun Tong, Sheng Cheng, Min Liu

**Affiliations:** 1 Department of Urology, Tongren Hospital, Shanghai Jiao Tong University School of Medicine, Shanghai 200336, China; czixuan2023@sjtu.edu.cn (Z.C.);; 2 Department of Traditional Chinese Medicine, Tongren Hospital, Shanghai Jiao Tong University School of Medicine, Shanghai 200336, China; 3 College of Medicine, Yanbian University, Yanji 133002, China; 4 Department of Anesthesiology and Surgery, Tongren Hospital, Shanghai Jiao Tong University School of Medicine, Shanghai 200336, China; 5 Hongqiao International Institute of Medicine, Tongren Hospital, Shanghai Jiao Tong University School of Medicine, Shanghai 200336, China

**Keywords:** renal cell carcinoma, esculin, natural products, network pharmacology, PI3K/Akt pathway

## Abstract

Purpose: This study aims to explore the potential mechanisms of esculin in the treatment of renal cell carcinoma (RCC). Methods: We employed network pharmacology to predict the potential mechanisms and targets of esculin in RCC. Molecular docking techniques were then employed to validate the predicted targets. Additionally, a series of in vitro experiments were conducted to verify the anticancer effects of esculin on RCC cells, including the CCK-8 assay, EdU assay, wound healing assay, apoptosis assay, and Western blot. Results: Network pharmacology and molecular docking results identified GAPDH, TNF, GSK3B, CCND1, MCL1, IL2, and CDK2 as core targets. GO and KEGG analyses suggested that esculin may influence apoptotic processes and target the PI3K/Akt pathway in RCC. Furthermore, the CCK-8 assay demonstrated that esculin inhibited RCC cell viability. Microscopic observations revealed that following esculin treatment, there was an increase in cell crumpling, a reduction in cell density, and an accumulation of floating dead cells. Additionally, with increasing esculin concentrations, the proportion of EdU-positive cells decreased, the wound closure ratio decreased, the proportion of PI-positive cells increased, the expression levels of BAX and cleaved-caspase-3 proteins increased, and the expression level of Bcl2 protein decreased. These findings suggested that esculin inhibits the proliferation and migration of RCC cells while promoting apoptosis. Moreover, esculin was found to target GAPDH and inhibit the PI3K/Akt pathway. Conclusions: This study is the first to elucidate the therapeutic effects of esculin on RCC cells. The results provide evidence supporting the clinical application of esculin and introduce a promising new candidate for RCC treatment.

## 1. Introduction

Renal cell carcinoma (RCC) accounts for 90% of all kidney tumors, making it the most common type of kidney cancer and the urological tumor with the highest mortality rate [1]. Surgical removal of the regional tumor is the most effective treatment approach for most RCC patients. However, approximately 30% of patients present with metastatic tumors at the time of RCC diagnosis, which limits the potential for surgical intervention [2]. The advent of immune checkpoint inhibitors and receptor tyrosine kinase inhibitors has significantly improved the survival rate of patients with advanced RCC [3]. Nevertheless, the emergence of resistance to chemotherapeutic agents remains a significant challenge in RCC treatment [4]. For instance, in the case of sunitinib, a receptor tyrosine kinase inhibitor used as a first-line treatment of RCC, resistance commonly develops within 6 to 15 months of treatment in the majority of patients [5]. Therefore, the continued investigation of novel agents to overcome resistance to current therapies is essential for improving the prognoses of patients with RCC.

In recent years, a growing body of research has demonstrated that active substances derived from natural products, such as plants, exhibit significant antitumor activity [6]. As of 2019, approximately half of the 247 new anticancer drugs approved by the United States Food and Drug Administration (FDA) were derived from natural products [7]. Esculin, an active substance extracted from the Chinese herb *Cortex Fraxini*, is chemically known as 6,7-dihydroxycoumarin. Esculin has demonstrated important medicinal properties in a wide range of diseases, including septic cardiomyopathy [8], osteoarthritis [9], acute lung injury [10], and liver fibrosis [11]. Additionally, the antitumor activity of esculin has been the focus of considerable research. For instance, Ji et al. [12] demonstrated that esculin inhibits colorectal carcinogenesis and progression by promoting ferroptosis. Similarly, Mokdad-Bzeouich et al. [13] showed that esculin inhibits migration and angiogenesis in glioblastoma. However, the potential efficacy of esculin in the treatment of RCC has not yet been investigated.

Network pharmacology is an analytical approach that integrates systems biology, bioinformatics, and pharmacology [14]. By analyzing the common targets of drug components and diseases and constructing complex network models, network pharmacology can reveal the interactions among drugs, proteins, and diseases [15]. This method is currently employed extensively to elucidate the mechanisms of action of natural products, including traditional Chinese medicine [16]. Peng et al. predicted that MYC, MAPK8, and CXCL8 might be the targets of Astragali Radix in acetaminophen (APAP)-induced liver injury (ALI) by using network pharmacology. Subsequently, the therapeutic efficacy of Astragali Radix in treating ALI was demonstrated through experimentation [17]. Zhang et al. verified that acteoside, an active ingredient in a natural product, can have a potential therapeutic effect on diabetic kidney disease by targeting the NF-κB pathway, using tools of network pharmacology such as Chinese medicine databases and molecular docking [18]. In addition to these, components of natural products such as Polygonati Rhizoma [19], Chuanbeimu [20], and β-sitosterol [21] have also been identified through network pharmacology for their roles in treating diseases. Network pharmacology has shown considerable potential in providing innovative strategies for disease treatment.

The objective of this study was to investigate the potential mechanism of esculin in the treatment of RCC. Initially, network pharmacology was employed to predict the interaction targets of esculin and RCC, as well as the biological functions of these targets. Molecular docking techniques were then applied to confirm the core targets within the intersecting targets of esculin and RCC. Finally, in vitro experiments were conducted to validate the antitumor effects and mechanism of esculin in RCC. This study expands the field of esculin’s role in disease treatment and presents a novel potential therapeutic strategy for the treatment of RCC.

## 2. Results

### 2.1. Network Pharmacology Predicts the Mechanisms of Esculin Treats RCC

**Figure 1:**
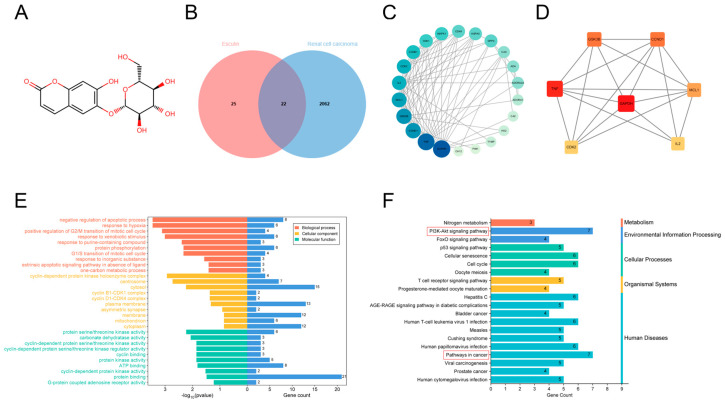
Network pharmacology analysis of esculin and RCC. (**A**) Chemical structure of esculin. (**B**) Venn diagram showing the intersecting genes between esculin and RCC. (**C**) PPI network of the intersecting genes between esculin and RCC. (**D**) Core protein targets between esculin and RCC. (**E**) GO analysis of the intersecting genes. (**F**) KEGG pathway analysis of the intersecting genes.

We first analyzed the 47 esculin targets identified from SwissTargetPrediction and the 2085 RCC targets obtained from the GeneCards database using Venn diagrams, identifying 22 intersecting genes (Supplementary Table S1) (Figure 1B). The PPI network data obtained from the STRING database were then analyzed using Cytoscape (Supplementary Table S2), with proteins displayed according to their degree. The larger and darker the protein node, the higher its degree (Figure 1C). Among these, GAPDH had the highest degree (degree of 16), while CA12 and PIM1 had the lowest (degree of 1). Based on degree ordering, the top seven proteins—glyceraldehyde-3-phosphate dehydrogenase (GAPDH), tumor necrosis factor (TNF), glycogen synthase kinase-3 beta (GSK3B), G1/S-specific cyclin-D1 (CCND1), myeloid cell leukemia-1 (MCL1), interleukin-2 (IL2), and cyclin-dependent kinase 2 (CDK2)—were identified as the core protein targets, which may be key targets for esculin in the treatment of RCC (Figure 1D).

Next, we used 22 intersecting genes to predict the potential mechanism of esculin in the treatment of RCC. GO analysis identified 83 BPs, 21 CCs, and 20 MFs, with the top 10 results visualized (Figure 1E). The results indicated that the BPs of these intersected genes were primarily associated with processes such as negative regulation of the apoptotic process, the extrinsic apoptotic signaling pathway in the absence of ligands, and protein phosphorylation. Significant MFs were related to protein serine/threonine kinase activity, ATP binding, and protein binding. The most significantly enriched CC was the cyclin-dependent protein kinase holoenzyme complex. KEGG pathway analysis identified a total of 64 pathways, with 20 of them visualized (Figure 1F). After categorizing the pathways, they were mainly found to be related to metabolism, environmental information processing, cellular processes, organismal systems, and human diseases. Notably, the pathways with the highest gene count were the PI3K-Akt signaling pathway and pathways in cancer.

### 2.2. Molecular Docking of Core Target Proteins with Esculin

The molecular docking technique can verify the reliability of the screened core target proteins by calculating the affinity of drug–protein binding. We docked esculin with seven core target proteins after dehydrogenating and hydrogenating the protein structures and hydrogenating the esculin structure. The results demonstrated that the affinity of esculin with all the proteins was less than 0, indicating successful binding to each of them.

**Figure 2:**
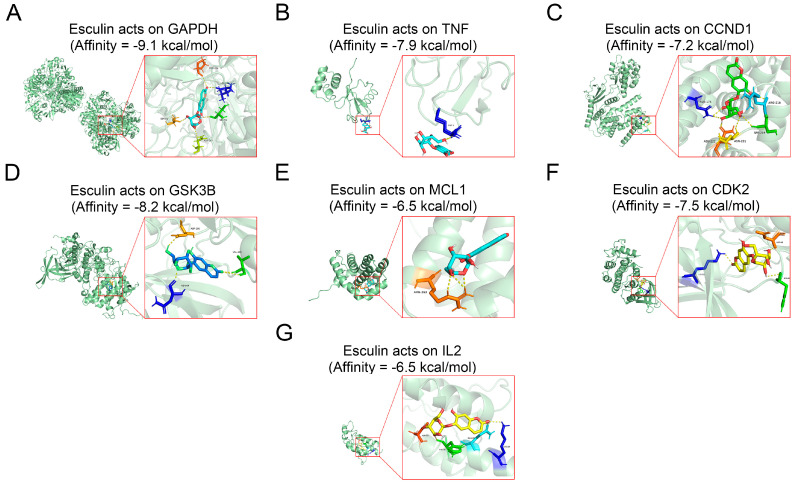
Molecular docking of core target proteins. (**A**) GAPDH; (**B**) TNF; (**C**) CCND1; (**D**) GSK3B; (**E**) MCL1; (**F**) CDK2; (**G**) IL2.

The binding energy of a drug molecule to a protein is determined by the strength of their interaction, with a lower binding energy indicating a stronger binding effect [22]. Among the proteins, GAPDH exhibited the lowest binding energy, with an affinity of −9.1 kcal/mol, suggesting that GAPDH may be the most central target of esculin in the treatment of RCC (Figure 2A) (Supplementary Table S3). The binding affinities for the other proteins were as follows: TNF, −7.9 kcal/mol (Figure 2B); CCND1, −7.2 kcal/mol (Figure 2C); GSK3B, −8.2 kcal/mol (Figure 2D); MCL1, −6.5 kcal/mol (Figure 2E); CDK2, −7.5 kcal/mol (Figure 2F); and IL2, −6.5 kcal/mol (Figure 2G). 

### 2.3. Esculin Inhibits RCC Cell Viability

**Figure 3:**
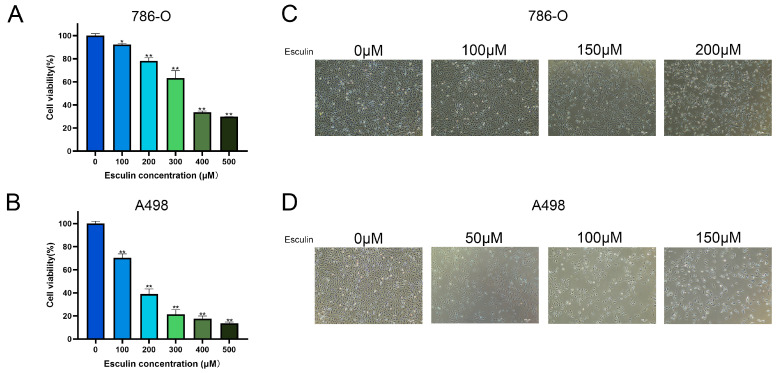
Effects of esculin on RCC cell viability. Cell viability of the RCC cells was assessed using the CCK-8 assay: (**A**) 786-O; (**B**) A498. Morphological changes in RCC cells after treatment with esculin: (**C**) 786-O; (**D**) A498. * *p* < 0.05, ** *p* < 0.01. Scale bar: 100 μm.

Following the application of network pharmacology, which predicted the potential therapeutic effects of esculin on RCC, we conducted a CCK-8 assay using RCC cell lines 786-O and A498 to investigate the inhibitory effects of esculin on RCC cells. The cells were treated with 0–500 μM esculin for 24 h, and a significant decrease in cell viability was observed with increasing esculin concentration. In 786-O, while cell viability was only mildly inhibited after 100 μM esculin treatment, with a cell viability of 92.28 ± 0.86%, it decreased to 78.12 ± 2.77% at 200 μM and to 63.30 ± 6.39% at 300 μM (Figure 3A). In A498, cell viability decreased to 70.38 ± 3.36% after treatment with 100 μM esculin and to 38.98 ± 4.35% at 200 μM (Figure 3B).

Subsequent observations of the cells under a light microscope in the bright field revealed that with increasing esculin concentrations, the cells exhibited a greater degree of crumpling, a reduction in cell density, and an increase in the number of floating dead cells (Figure 3C,D).

These combined results demonstrated that esculin is capable of inhibiting the viability of renal cancer cells. In the range of 0 μM to 50 μM, the degree of inhibition enhances with increasing concentration.

### 2.4. Esculin Inhibits RCC Cell Proliferation and Migration

**Figure 4:**
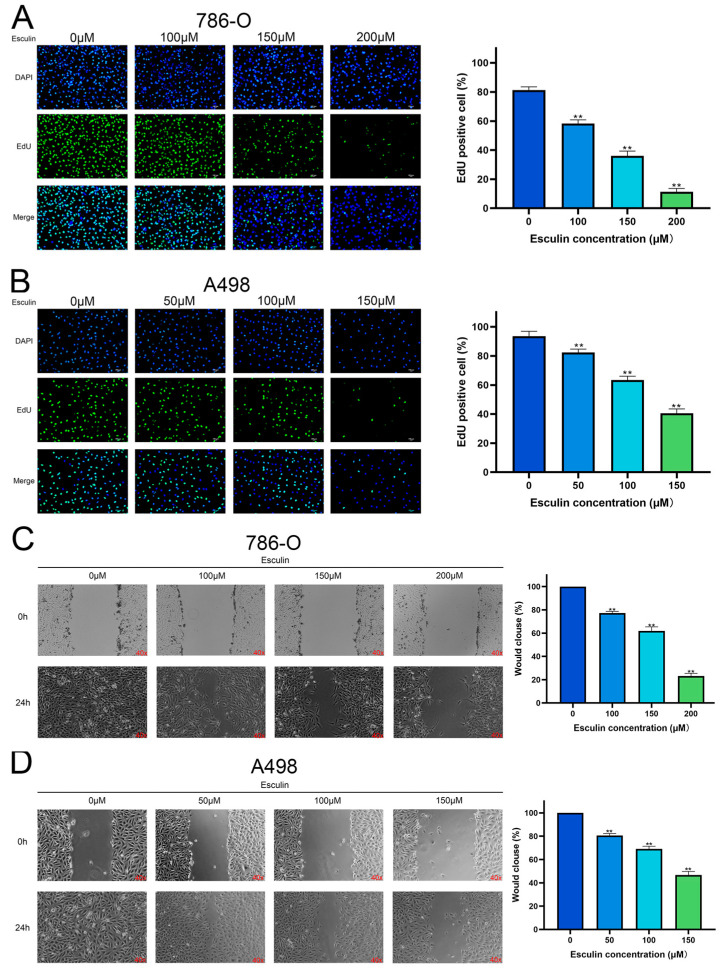
Effects of esculin on RCC cell proliferation and migration. Esculin inhibits the proliferation of RCC cells using the EdU assay with blue (DAPI) and green (EdU): (**A**) 786-O; (**B**) A498. Scale bar: 100 μm. Esculin inhibits the migration of RCC cells using the wound healing assay: (**C**) 786-O; (**D**) A498. ** *p* < 0.01.

We further verified the inhibitory effect of esculin on RCC cells by employing EdU and wound healing assays to investigate its effects on the proliferation and migration of 786-O and A498 cells, respectively. The results of the EdU assay demonstrated that in 786-O, the percentage of EdU-positive cells decreased from 81.31 ± 2.20% to 58.37 ± 2.46% with 100 μM esculin, and it further dropped to 11.26 ± 2.33% with 200 μM esculin (Figure 4A). Similar results were observed in A498, where higher esculin concentrations resulted in lower EdU-positive cell ratios. The control group exhibited an EdU-positive cell ratio of 93.55 ± 3.35%, which was reduced to 40.56 ± 2.95% with 150 μM esculin (Figure 4B). These findings indicated that esculin treatment reduces the proliferation of RCC cells compared to the control group.

The wound healing assay revealed that in the absence of esculin, wound closure was complete after 24 h in both 786-O and A498 cells. However, the presence of esculin led to a dose-dependent decrease in the rate of wound closure. Specifically, the addition of 100 μM, 150 μM, and 200μM esculin reduced the closure rate of 786-O to 77.21 ± 1.52%, 61.89 ± 3.52%, and 23.08 ± 2.45%, respectively (Figure 4C). In A498, the closure rate decreased to 80.63 ± 1.68%, 69.17 ± 2.22%, and 46.75 ± 3.03% with 50 μM, 100 μM, and 150 μM esculin, respectively (Figure 4D). These results demonstrated that esculin inhibits the migration of RCC cells in a dose-dependent manner.

### 2.5. Esculin Induces Apoptosis in RCC Cells

**Figure 5:**
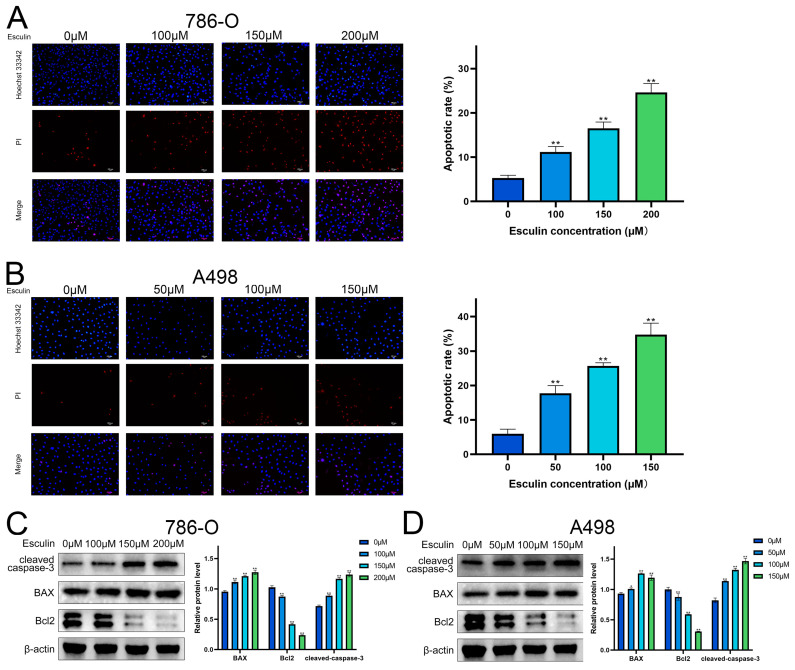
Effects of Esculin on apoptosis of RCC cells. Hoechst 33342/PI (blue/red) co-staining to detect apoptosis in RCC cells after treatment with esculin: (**A**) 786-O; (**B**) A498. Scale bar: 100 μm. Images and quantification of Western blot of cleaved-caspase-3, BAX, Bcl2 after treatment with esculin: (**C**) 786-O; (**D**) A498. * *p* < 0.05, ** *p* < 0.01.

GO analysis suggested a potential link between esculin and RCC through the induction of apoptosis. Consequently, we investigated the impact of esculin on apoptosis in RCC cells. Propidium iodide (PI) is a cytosolic dye that binds to DNA and emits red fluorescence when apoptosis occurs due to the disruption of cell membrane integrity [23]. A comparison of the ratio of PI-positive cells revealed that esculin treatment led to an increase in the apoptosis rate in RCC cells. As the concentration of esculin increased, the apoptosis rate of 786-O cells rose from 5.30 ± 0.62% to 24.65 ± 1.96% (Figure 5A), while in A498, it increased from 5.96 ± 1.35% to 34.74 ± 3.35% (Figure 5B).

We also investigated the impact of esculin on apoptosis in RCC cells at the protein level. BAX and Bcl2 are two key proteins that regulate apoptosis, with BAX acting as a pro-apoptotic factor and Bcl2 as an anti-apoptotic factor. During apoptosis, the BAX/Bcl2 ratio increases. Caspase-3, a cysteine–aspartate protease, is widely used as an apoptotic marker, and it is cleaved to form cleaved caspase-3 during apoptosis [24]. Western blotting showed that esculin treatment resulted in increased protein expression levels of cleaved caspase-3 and BAX, and decreased protein levels of Bcl2 in both A498 and 786-O (Figure 5C,D).

These experimental results demonstrated that esculin can induce apoptosis in RCC cells.

### 2.6. Esculin Inhibits RCC Cells via PI3K/Akt Pathway

**Figure 6:**
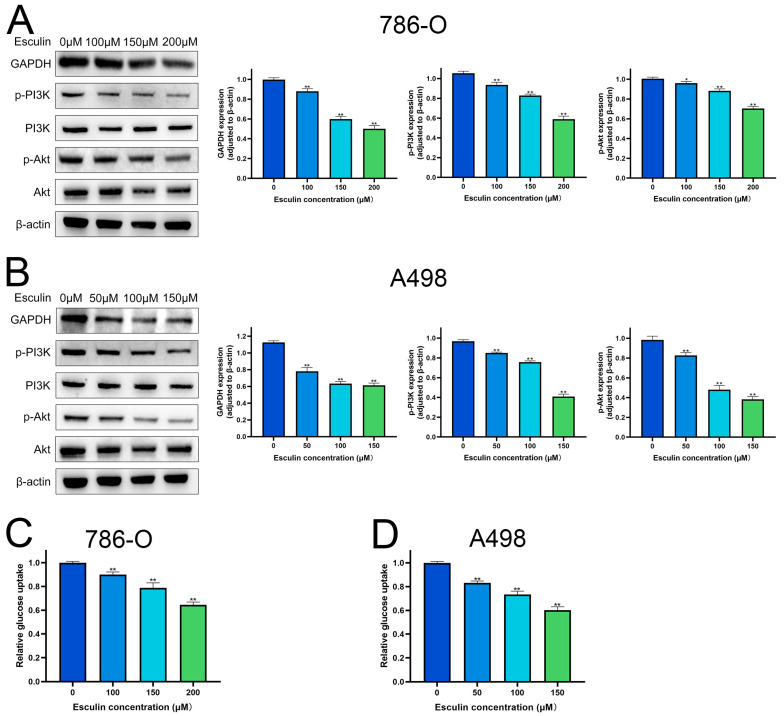
Effects of esculin on PI3K/Akt pathway and glucose metabolism in RCC cells. Images and quantification of Western blot of GAPDH, p-PI3K and p-Akt after treatment with esculin: (**A**) 786-O; (**B**) A498. Glucose uptake of RCC cells: (**C**) 786-O; (**D**) A498. * *p* < 0.05, ** *p* < 0.01.

Molecular docking results identified GAPDH as the most central target of esculin’s action on RCC. Therefore, we first explored the effect of esculin on GAPDH expression. Western blotting revealed that the expression level of GAPDH was significantly downregulated in RCC cells following esculin treatment. Additionally, we verified the pathway through which esculin exerts its effects in RCC. KEGG analysis suggested that esculin may act through the PI3K/Akt pathway. To confirm this, we used Western blotting to examine the changes in the protein levels of p-PI3K and p-Akt. The results showed that the expression levels of p-PI3K and p-Akt were significantly decreased in both 786-O and A498 after esculin treatment (Figure 6A,B). These findings indicated that esculin may inhibit RCC by downregulating the PI3K/Akt pathway. 

GAPDH is a glycolytic enzyme crucial for glucose metabolism, and its inhibition can disrupt glycolysis [25]. Western blot analysis confirmed that esculin inhibits GAPDH expression. Consequently, we investigated esculin’s impact on glucose metabolism. The results revealed that increased concentrations of esculin significantly reduced glucose uptake in 786-O and A498 (Figure 6C,D), indicating that esculin affects glucose metabolism in RCC cells.

## 3. Discussion

Kidney cancer is associated with extremely high morbidity and mortality, with approximately 400,000 new cases diagnosed and 175,000 deaths reported worldwide each year [26]. RCC is the most common type of kidney cancer. Due to its tendency for progression and metastasis, immunotherapy has become a crucial approach in the treatment of RCC [27]. However, the emergence of resistance to immunotherapy has necessitated the ongoing investigation of novel drugs to address the challenge of drug resistance in RCC [28].

Natural products typically comprise a wide range of active substances, including saponins, alkaloids, flavonoids, and lignans [29]. In recent years, an increasing number of studies have substantiated the potential of natural products, or specific substances within them, for cancer treatment. This is largely due to their capacity to inhibit cancer development by affecting cell proliferation, angiogenesis, and other biological processes within tumors [30]. Natural products also play an important role in the treatment of RCC. For instance, thymoquinone, as a compound in *Nigella Sativa*, not only has renal protective effects but also inhibits RCC [31]. Similarly, arborinine, an extract from the *Globigerina parva leaf*, was found by Feng et al. to control the progression of RCC by inhibiting epithelial mesenchymal transition [32]. In addition, natural products such as genistein, kahweol acetate, and sinularin have demonstrated antitumor effects in RCC treatment through various mechanisms, including promoting apoptosis, inhibiting angiogenesis, and increasing chemosensitivity [33]. Esculin, a natural product with known protective effects on the kidney [34], has not yet been investigated for its potential role in RCC treatment. This lack of evidence prompted us to explore the inhibitory effect of esculin on RCC in this study.

Network pharmacology, an emerging multidisciplinary research method, offers innovative approaches for studying natural product medicine. It can predict the targets of natural products or their components in diseases, construct interaction networks of these targets, and ultimately reveal the potential therapeutic mechanisms of natural products [35]. A growing number of studies have leveraged network pharmacology to explore the medicinal value of various natural products. Similarly, we applied network pharmacology to predict the potential mechanism of esculin in the treatment of RCC. Our analysis identified 22 intersecting genes between esculin and RCC, primarily related to BPs such as apoptosis and pathways such as cancer and PI3K/Akt. A PPI network was constructed, and the degree of interaction among the intersecting genes was analyzed, leading to the identification of seven core target genes (degree ≥ 10): GAPDH, TNF, GSK3B, CCND1, MCL1, IL2, and CDK2. We then used molecular docking technology to analyze the affinity and predicted the amino acid residues where esculin binds to these proteins. Our findings indicated that esculin could bind to all these proteins. Specifically, esculin binds to GAPDH via the binding sites SER-51, ASN-287, LEU-203, and PRO-236; to TNF via MET-1; to CCND1 via ASN-174, ARG-218, SER-219, and ASN-211/222; to GSK3B via ASN-64, ASP-200, and VAL-135; to MCL1 via ARG-263; to CDK2 via LYS-33, HIS-84, and ASP-86; and to IL2 via ASP-40, HIS-36, GLN-33, and LYS-29. Among these, GAPDH exhibited the lowest binding affinity with esculin, with an affinity of −9.1 kcal/mol. Therefore, we hypothesized that GAPDH is the most critical target for esculin in the treatment of RCC.

GAPDH is not only a key enzyme in the glycolytic pathway but also plays a significant role in the development and treatment of cancer [36]. Studies have shown that GAPDH is upregulated in various cancers and is associated with poor prognosis [37,38]. The impact of GAPDH on cancer is primarily observed in two aspects. On one hand, GAPDH participates in the glycolytic process, providing essential energy and substrates for cellular activities such as the proliferation, invasion, and metastasis of cancer cells, ultimately promoting cancer growth [39]. On the other hand, GAPDH helps cancer adapt to harsh environments by regulating oxidative stress responses and apoptosis, thereby facilitating cancer progression [40]. Based on this, targeting GAPDH to block the energy and substrate supply to cancer cells or to induce apoptosis has garnered significant attention as an anticancer strategy. For example, DC-5163, a GAPDH inhibitor, effectively treats breast cancer by reducing energy supply through the inhibition of glycolysis [41]. Similarly, AXP-3019, a 3-bromo-isoxazoline derivative, inhibits the proliferation of pancreatic cancer cells by targeting and inhibiting GAPDH [42]. In our study, Western blotting also revealed that esculin inhibits the expression of GAPDH and reduces glucose uptake in RCC cells, thereby affecting glucose metabolism.

After using network pharmacology to predict that esculin may have a therapeutic effect on RCC, we conducted a series of in vitro experiments for validation. First, the CCK-8 assay was applied to assess the inhibition of RCC cell viability by esculin. The results demonstrated that esculin inhibited cell viability in a dose-dependent manner. Interestingly, we observed differential IC50 values for esculin in the two RCC cell lines. Specifically, the IC50 for Esculin was 339.9 μM in 786-O cells and 158.0 μM in A498 cells. We believe that this phenomenon is due to cellular heterogeneity, which results in different effective concentrations of the same drug across various cell lines within the same disease [43]. This finding was further supported by microscopic observation of cellular morphology. Next, we used the EdU assay and wound healing assay to verify the inhibitory effects of esculin on the proliferation and migration of RCC cells. The results showed that as the concentration of esculin increased, there was a corresponding increase in the inhibition of cell proliferation and migration, compared to the control group. For apoptosis detection, we employed both Hoechst 33342/PI co-staining and Western blot analysis. The fluorescence imaging results demonstrated that higher concentrations of esculin were associated with a greater ratio of PI-positive cells and a higher degree of apoptosis. Additionally, Western blot revealed that the expression of the pro-apoptotic factor BAX was elevated, the anti-apoptotic factor Bcl2 was decreased, and the apoptotic marker cleaved-caspase-3 was gradually increased. These findings indicated that esculin induces apoptosis in RCC cells. Finally, we employed Western blotting to assess the expression levels of p-PI3K and p-Akt in RCC cells following esculin treatment. The results were consistent with KEGG pathway analysis predictions, showing that esculin downregulated these two proteins, thereby demonstrating targeted inhibition of the PI3K/Akt pathway.

The PI3K/Akt pathway plays a critical role in the development of cancer and the emergence of chemotherapeutic drug resistance, making it a focal point of research in various cancers, including RCC [44]. Guo et al. found that alpinetin inhibits the proliferation and migration of RCC by targeting the PI3K/Akt/mTOR pathway [45]. Similarly, Xi et al. demonstrated that homoharringtonine effectively controls thyroid cancer progression by inhibiting the TIMP1/FAK/PI3K/AKT pathway [46]. Wei et al. revealed that Lianhua Qingwen exhibits synergistic effects with sorafenib in the treatment of liver cancer by targeting the PI3K/Akt pathway [47]. These studies suggest that targeting the PI3K/Akt pathway could be an important therapeutic strategy for cancer treatment. Our study, through KEGG analysis and Western blotting, also confirmed that esculin inhibits RCC cells by targeting this crucial pathway.

Interestingly, our molecular docking results indicated that GAPDH could serve as a critical target for esculin in the treatment of RCC. Moreover, the interaction between GAPDH and Akt has been well-documented. Jacquin and colleagues demonstrated that GAPDH can bind to Akt, thereby inhibiting Akt phosphorylation and regulating the PI3K/Akt pathway [48]. Additionally, Zhang et al. found that inhibition of GAPDH could suppress tumor cell genesis and proliferation by downregulating the expression of p-Akt [36]. Consistent with previous findings, our study also found that esculin can target GAPDH and inhibit the PI3K/Akt pathway. This provides solid evidence that GAPDH is the core target of esculin.

However, our study has some limitations. We demonstrated the inhibitory effect of esculin on RCC through in vitro experiments, but further in vivo experimental validation is necessary. Additionally, while molecular docking analysis suggested that GAPDH might be a key target of esculin for RCC—and we demonstrated that esculin can inhibit the expression of GAPDH, is associated with the PI3K/Akt pathway, and also inhibits glucose uptake—further investigation is needed to determine whether esculin can regulate GAPDH to influence the occurrence and progression of RCC. Moreover, esculin is a component of *Cortex Fraxini*, a commonly used traditional Chinese medicine that contains various active ingredients with a wide range of pharmacological activities, including caffeic acid, tyrosol, and scopoletin, all of which also exhibit antitumor activity [49]. For example, caffeic acid has been shown to be a potent antitumor agent, effectively inhibiting liver, breast, and skin cancer [50]. Tyrosol derivatives inhibit leukemia by promoting apoptosis [51]. Similar to esculin, scopoletin inhibits non-small-cell lung cancer by targeting the PI3K/Akt pathway [52]. Therefore, whether esculin and other components of *Cortex Fraxini* could have synergistic effects in antitumor action, thereby enhancing their therapeutic effect on RCC, deserves further exploration.

## 4. Materials and Methods

### 4.1. Reagents

Reagents were sourced as follows: esculin (T2885, TargetMol, Shanghai, China), CCK-8 (SB-CCK8, Share-bio, Shanghai, China), EdU Imaging Kits (K2240, APExBIO, Houston, TX, USA), Hoechst 33342/PI Double Staining Kit (K2237, APExBIO, Houston, TX, USA), DAPI (abs47047616, Absin, Shanghai, China), Cell Freezing Medium (211072, NEST, Wuxi, China), Screen Quest™ Colorimetric Glucose Uptake Assay Kit (36503, AAT Bioquest, Sunnyvale, CA, USA), RIPA lysis buffer (PC101, EpiZyme, Shanghai, China), protease inhibitor cocktail and phosphatase inhibitor cocktail (GRF101/GRF102, EpiZyme, China), BCA assay kit (SB-WB013, Share-bio, Shanghai, China), Bis-Tris pre-cast gels (SB-FP11010, Share-bio, Shanghai, China), anti-BAX (1:1000, 50599-2-lg, Proteintech, Wuhan, China), anti-Bcl2 (1:1000, 68103-1-lg, Proteintech, Wuhan, China), anti-cleaved-caspase-3 (1:1000, 9661, CST, Beverly, MA, USA), anti-p-PI3K (1:1000, 4228, CST, Beverly, MA, USA), anti-PI3K (1:1000, 4292, CST, Beverly, MA, USA), anti-p-Akt (1:1000, 13038, CST, Beverly, MA, USA), anti-Akt (1:1000, 4691, CST, Beverly, MA, USA), anti-β-actin (1:9000, ZB15001-HRP-100, Servicebio, Wuhan, China), and HRP-conjugated secondary antibodies (1:2000, 7074S/7076S, CST, Beverly, MA, USA).

### 4.2. Targets Acquisition and Analysis

Esculin targets were obtained from SwissTargetPrediction (http://www.swisstargetprediction.ch/index.php) (accessed on 11 August 2024). First, the isomeric SMILES of esculin were retrieved from PubChem (https://pubchem.ncbi.nlm.nih.gov/) (accessed on 11 August 2024). On the SwissTargetPrediction website, select “Homo sapiens”, enter the isomeric SMILES, and click “Predict targets”. Finally, export the analyzed targets. Isomeric SMILES: C1=CC(=O)OC2=CC(=C(C=C21)O[C@H]3[C@@H]([C@H]([C@@H]([C@H](O3)CO)O)O)O)O. RCC targets were obtained from GeneCards (https://www.genecards.org) (accessed on 11 August 2024) by searching for “Renal cell carcinoma” and saving the relevant disease genes. 

The intersecting targets of esculin and RCC were analyzed using the jvenn tool in bioinformatics [53]. The intersecting genes were identified by entering the targets of esculin and RCC separately into the tool.

### 4.3. PPI Network Construction

The STRING database facilitates the analysis of protein interaction relationships. The intersecting targets of esculin and RCC were entered into STRING to obtain a protein-protein interaction (PPI) network diagram. The Cytoscape 3.9.1 software was then used to analyze and visualize the degree between targets, and the top 7 targets based on degree were identified as core targets.

### 4.4. Analysis of GO and KEGG Pathway

Gene Ontology (GO) and Kyoto Encyclopedia of Genes and Genomes (KEGG) pathway analyses were conducted using the Database for Annotation, Visualization, and Integrated Discovery (DAVID) (https://david.ncifcrf.gov/) (accessed on 11 August 2024). The intersecting targets were entered into the DAVID website and the data for biological processes (BP), cellular components (CC), molecular functions (MF), and KEGG pathways were downloaded from the analysis results. Finally, the results were visualized using bioinformatics.

### 4.5. Molecular Docking

Seven core protein targets were selected for docking with esculin. The molecular structure of esculin was obtained from the TCMSP database (https://old.tcmsp-e.com/tcmsp.php) (accessed on 11 August 2024), and the 3D structures of the proteins were downloaded from the RCSB database (https://www.rcsb.org/) (accessed on 11 August 2024). The structures of esculin and proteins were processed by deleting water molecules and adding hydrogens using the AutoDockTools 1.5.7 software. Molecular docking and affinity calculations between esculin and the proteins were then performed using AutoDock 1.2.0 Vina. Visualization of the docking results was conducted using the PyMOL 2.6 software.

### 4.6. Cell Lines

The human renal cell carcinoma cell lines 786-O and A498 were purchased from Zhong Qiao Xin Zhou Biotechnology (Shanghai, China). The 786-O cells were cultured in RPMI-1640 complete medium (ZQXZbio, Shanghai, China), while the A498 cells were cultured in EMEM complete medium (ZQXZbio, Shanghai, China). Both cell lines were maintained in an incubator at 37 °C with 5% CO_2_.

### 4.7. CCK-8 Assay

786-O and A498 cells were inoculated into 96-well plates at a density of 5000 cells/well. After the cells adhered, different concentrations of esculin were added to the wells. Following 24 h of esculin treatment, 10 µL CCK-8 was added to each well. The plates were then incubated at 37 °C for 1 h. The absorbance of each well was measured at 450 nm using a Multiskan FC Microplate Reader (Thermo Scientific, Waltham, MA, USA). Cell viability was calculated using the formula: Cell viability (%) = [(OD value of experimental well − OD value of blank well)/(OD value of control well − OD value of blank well)] × 100%. The IC50 values were calculated using GraphPad Prism 9.0.

### 4.8. EdU Assay

786-O and A498 cells were inoculated into 96-well plates at an appropriate density. After the cells adhered, different concentrations of esculin were added, and the cells were treated for 24 h. Following this treatment, 10 μM EdU was added to each well, and incubation continued for another 24 h. Subsequently, cell fixation, permeabilization, and staining were performed according to the instructions provided with the EdU detection kit. Images of green fluorescence (EdU) and blue fluorescence (DAPI) were captured using a fluorescence microscope. The number of EdU-positive cells and DAPI-stained cells were counted using the Image J 1.54 software, and the percentage of EdU-positive cells was calculated.

### 4.9. Wound Healing Assay

786-O and A498 cells were inoculated into 6-well plates. Once the cells reached confluence, wounds were created using a pipette tip, and the initial wound areas were photographed under a microscope. Different concentrations of esculin were then added to the wells for a 24 h treatment period. After treatment, the wound areas were photographed again to document changes. The wound areas at both time points were measured using the Image J 1.54 software, and the wound closure rates were calculated.

### 4.10. Apoptosis Assay

786-O and A498 cells were inoculated into 96-well plates. After the cells adhered, different concentrations of esculin were added, and the cells were treated for 24 h. Following the treatment, staining was performed according to the protocol of the Hoechst 33342/PI co-staining kit. Fluorescence images were captured using a fluorescence microscope. The number of PI-positive cells was counted using the Image J 1.54 software, and the apoptosis rate was calculated.

### 4.11. Glucose Uptake Assay

786-O and A498 cells were inoculated into 96-well plates and treated with varying concentrations of esculin. After treatment, 2-DG solution was added to the wells following the manufacturer’s instructions, and the cells were incubated at 37 °C for 30 min. The cells were then washed and lysed, and 50 μL of 2-DG Uptake Assay working solution was added to each well. The plates were incubated in the dark at room temperature for 1 h. Absorbance was measured at 570 nm.

### 4.12. Western Blot

Cells were lysed using RIPA lysis buffer containing protease and phosphatase inhibitors to extract proteins. Protein concentration was quantified using a BCA assay kit. The proteins were separated on pre-cast gels and transferred to a PVDF membrane using a rapid transfer buffer. The membrane was blocked with a rapid blocking buffer and incubated overnight at 4 °C with primary antibodies. After washing the membrane three times with TBST, it was incubated with secondary antibodies at room temperature for 1 h, followed by three additional washes with TBST. The blots were then visualized using a Tanon-5200 chemiluminescence imaging system (Tanon Science & Technology, Shanghai, China). Finally, the grayscale values were analyzed using the Image J software. Original figures can be found in Supplementary Materials. 

### 4.13. Statistical Analysis

Unless otherwise specified, all experiments were conducted in triplicate. Data analysis and visualization were performed using GraphPad Prism 9.0. Statistical differences among experimental groups were analyzed using one-way analysis of variance (ANOVA). A *p*-value < 0.05 was considered statistically significant.

## 5. Conclusions

In summary, esculin inhibited cell viability, cell proliferation, and cell migration in RCC cells. Esculin upregulated the PI-positive cell rate, promoted the expression of BAX and cleaved caspase-3, and inhibited the expression of Bcl2 in RCC cells. This therapeutic effect may be achieved by targeting the PI3K/Akt pathway. This research expands the application of esculin in cancer treatment and provides a novel strategy for the therapy of RCC.

## Data Availability

All new data have been presented in this paper. There are no further data, but the author welcomes questions and discussion.

## Supplementary Materials

The following supporting information can be downloaded at: https://www.mdpi.com/article/10.3390/biom14081043/s1, Table S1: The intersecting targets of Esculin and RCC; Table S2: The STING interactions between intersecting genes; Table S3: The amino acid residue sites of Esculin binds to core target proteins. Figure S1: Original Images for Blots.
