# Supplementary figures and images for: Revealing the Mechanism of Esculin in Treating Renal Cell Carcinoma Based on Network Pharmacology and Experimental Validation
Source: Biomolecules. 2024 Aug 22;14(8):1043. doi: 10.3390/biom14081043 (PMC11352311; doi:10.3390/biom14081043)

Figure S1: Original Images for Blots.

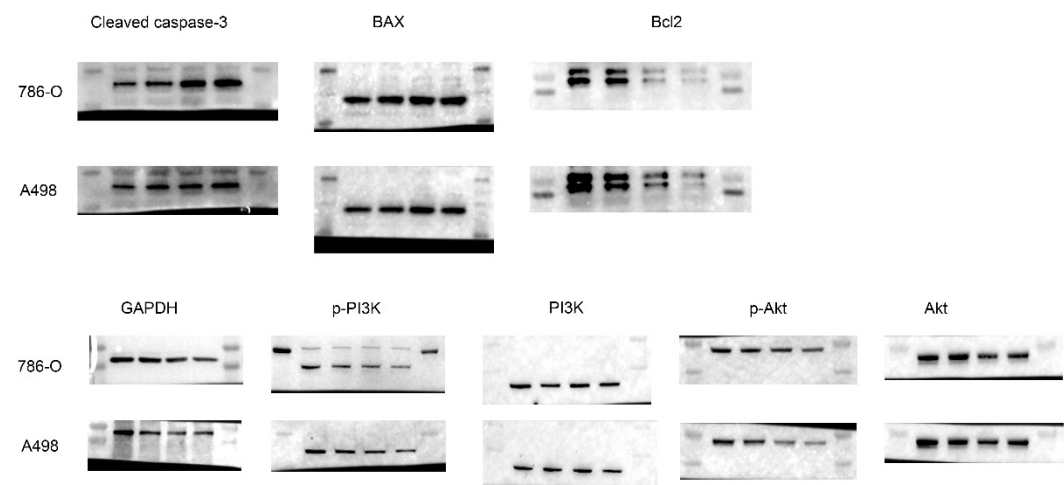

Supplement: Supplementary file 1 [file biomolecules-14-01043-s001.zip › Figure S1 Original Images for Blots..pdf]
